# Surprising finding of right-to-left shunt revealed with computed tomography angiography

**DOI:** 10.1007/s12471-020-01401-5

**Published:** 2020-03-10

**Authors:** M. Kardos, M. Sagat, M. Kaldararova, P. Tittel, M. Nosal

**Affiliations:** 1 Department of Functional Diagnostics, Children’s Cardiac Center, Bratislava, Slovakia; 2 Department of Cardiac Surgery, Children’s Cardiac Center, Bratislava, Slovakia

A 2-year-old boy presented to our inpatient department with a history of recurrent lower respiratory tract infection and central cyanosis. His systemic saturation was between 75–80%. Transthoracic echocardiography revealed no structural heart defect except for massive flow via dilated right pulmonary vein. Performed computed tomography angiography confirmed presence of this type of anomaly (Fig. 1). What is the diagnosis?

**Fig. 1:**
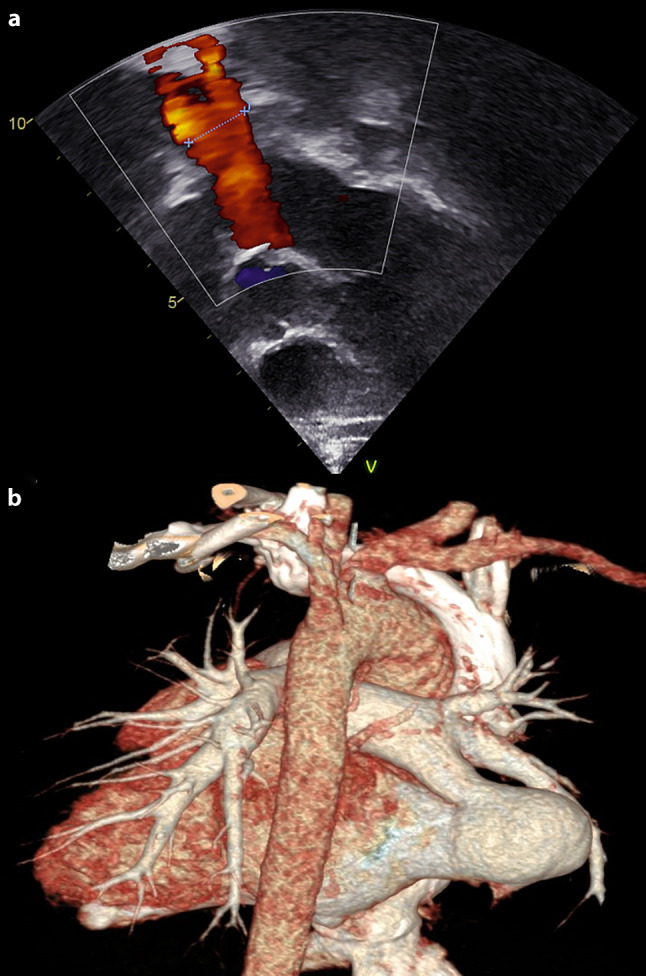
**a** Colour Doppler study showing massive pulmonary vein flow from the right pulmonary vein. **b** Volume rendering technique reconstruction of performed computed tomography angiography with surprising finding

## Answer

You will find the answer elsewhere in this issue.

